# Differentiation Generates Paracrine Cell Pairs That Maintain Basaloid Mouse Mammary Tumors: Proof of Concept

**DOI:** 10.1371/journal.pone.0019310

**Published:** 2011-04-26

**Authors:** Soyoung Kim, Shruti Goel, Caroline M. Alexander

**Affiliations:** McArdle Laboratory for Cancer Research, University of Wisconsin–Madison, Madison, Wisconsin; Sanford-Burnham Medical Research Institute, United States of America

## Abstract

There is a paradox offered up by the cancer stem cell hypothesis. How are the mixed populations that are characteristic of heterogeneous solid tumors maintained at constant proportion, given their high, and different, mitotic indices? In this study, we evaluate a well-characterized mouse model of human basaloid tumors (induced by the oncogene Wnt1), which comprise mixed populations of mammary epithelial cells resembling their normal basal and luminal counterparts. We show that these cell types are substantially inter-dependent, since the MMTV LTR drives expression of Wnt1 ligand in luminal cells, whereas the functional Wnt1-responsive receptor (Lrp5) is expressed by basal cells, and both molecules are necessary for tumor growth. There is a robust tumor initiating activity (tumor stem cell) in the basal cell population, which is associated with the ability to differentiate into luminal and basal cells, to regenerate the oncogenic paracrine signaling cell pair. However, we found an additional tumor stem cell activity in the luminal cell population. Knowing that tumors depend upon Wnt1-Lrp5, we hypothesized that this stem cell must express Lrp5, and found that indeed, all the stem cell activity could be retrieved from the Lrp5-positive cell population. Interestingly, this reflects post-transcriptional acquisition of Lrp5 protein expression in luminal cells. Furthermore, this plasticity of molecular expression is reflected in plasticity of cell fate determination. Thus, *in vitro*, Wnt1-expressing luminal cells retro-differentiate to basal cell types, and *in vivo*, tumors initiated with pure luminal cells reconstitute a robust basal cell subpopulation that is indistinguishable from the populations initiated by pure basal cells. We propose this is an important proof of concept, demonstrating that bipotential tumor stem cells are essential in tumors where oncogenic ligand-receptor pairs are separated into different cell types, and suggesting that Wnt-induced molecular and fate plasticity can close paracrine loops that are usually separated into distinct cell types.

## Introduction

There are various potential explanations for the cellular heterogeneity that exists in some tumors. Heterogeneity could reflect random allocation to different cell fates, or be the result of uncontrolled growth of a tumor stem cell, if that tumor stem cell is a normal stem cell “gone bad” [1]. In this scenario (given differentiation is not suppressed), the diversity of cell types existing in the tissue of origin could be recapitulated in the tumor. This could imply that differentiation is a passive process that has little functional significance for tumor growth. However, using a mouse model of basaloid tumors, we have noticed that differentiation to basal and luminal cell fates is important to the establishment of an oncogenic paracrine cell pair, and propose that this may be true also for human breast cancer patients.

Human and mouse basaloid breast tumors are characterized by the over-representation of mRNA species typically associated with basal cells in transcriptional profiles (for example, cytokeratins 5/6/14/17, TRIM29 and collagen type XVII). This often, but not always, correlates with the so-called “triple negative” status (ERα-, PR- and erbB2-negative) [2], [3], [4]. This is important from a therapeutic viewpoint, since these tumors are not good candidates for treatment with anti-ER or anti-erbB2 strategies. However, their molecular etiology is not well understood, and probably includes a number of distinct origins. Human basaloid breast tumors are aggressive, have high mitotic indices, and at least a proportion of this group of tumors maintains their cellular diversity during the entire disease course [5], [6]. They contain mammary epithelial cell types that resemble their basal and luminal cell counterparts in normal mammary gland, and are illustrated in tumor sections using immunohistochemistry [7].

Several classes of breast tumors that develop in mouse models cluster into a basaloid sub-class, including tumors that develop in response to the proto-oncogene, Wnt1 [8]. In preneoplastic mammary glands from [MMTV-LTR-Wnt1] transgenic mice, stem cells accumulate (defined by a functional assay of fat pad colonization at limiting dilutions) [9], [10]. The solitary tumors that arise in these hyperplastic glands comprise mixed populations of basal and luminal cells; when transferred at limiting cell dilutions, tumor cell populations robustly regenerate mixed populations [11], [12]. The formation and maintenance of these tumors depends upon the expression of Wnt1 ligand; thus, in mice that conditionally express Wnt1, tumors regress upon withdrawal of Wnt1 ligand expression [13]. Wnt signaling is complex, and various responses are mediated by a number of cell surface receptor species. However the principle tumorigenic Wnt signaling pathway is the so-called canonical pathway, mediated by the interaction of Frizzled-Lrp cell surface co-receptors [14]. We have previously shown that Lrp5 is necessary for tumor development in response to Wnt1 [15], despite the co-expression of functional Lrp6 in basal mammary epithelial cells [16]. Wnt signaling (via Lrp5 and Wnt1) is therefore necessary and sufficient for cell growth and tumor maintenance.

For mouse mammary gland, normal stem cells co-purify with basal cells (using flow cytometry with various cell surface markers) [17], [18]. When these cells are transferred *in vivo* to fat pads, they can recreate normal ductal trees, comprising both luminal and basal cells, and they show the same bipotentiality *in vitro*. Normal mammary stem cells are maintained by Wnt signaling, and depend upon Lrp5, together with an as yet unidentified Wnt ligand [16], [19].

Using this model system, we present a model to explain the co-existing cell populations, We propose that the distinct cell types interact to propagate the tumor, and that differentiation is required to promote the expression of the ligand. Bipotential stem cells are required to create optimal proportions of basal and luminal cells, to promote the growth of tumors that depend upon a paracrine interaction. This is a proof of concept that we propose could offer a discovery platform for therapeutic development, assuming that co-mingling of basal and luminal cell types could also have functional significance for human basaloid tumors. Specifically, we show that the Wnt1 ligand is expressed in luminal cells and the cognate receptor, Lrp5, is expressed by basal cells. Tumor initiating cells exist in the basal population, which can differentiate to regenerate the basal-luminal cell pair that drives growth. Since we also observe luminal cells that can serve as tumor-initiating cells, we tested the hypothesis that these cells might also express Lrp5 protein. Flow cytometric analysis showed the acquisition of low levels of cell surface Lrp5 by a luminal subpopulation, and indeed all the tumor stem cell activity co-purified with the Lrp5-positive cell population. Wnt1-expressing luminal cells not only showed molecular plasticity, they showed plasticity of cell fate *in vitro*, and *in vivo*. Thus, when used to seed a new tumor, they divide and differentiate to recreate typical basal-luminal tumor cell populations. We suggest this illustrates the selection pressure that maintains these heterogeneous tumors.

## Results

### Separation of luminal and basal cells from MMTV-Wnt1 tumors

**Figure 1:**
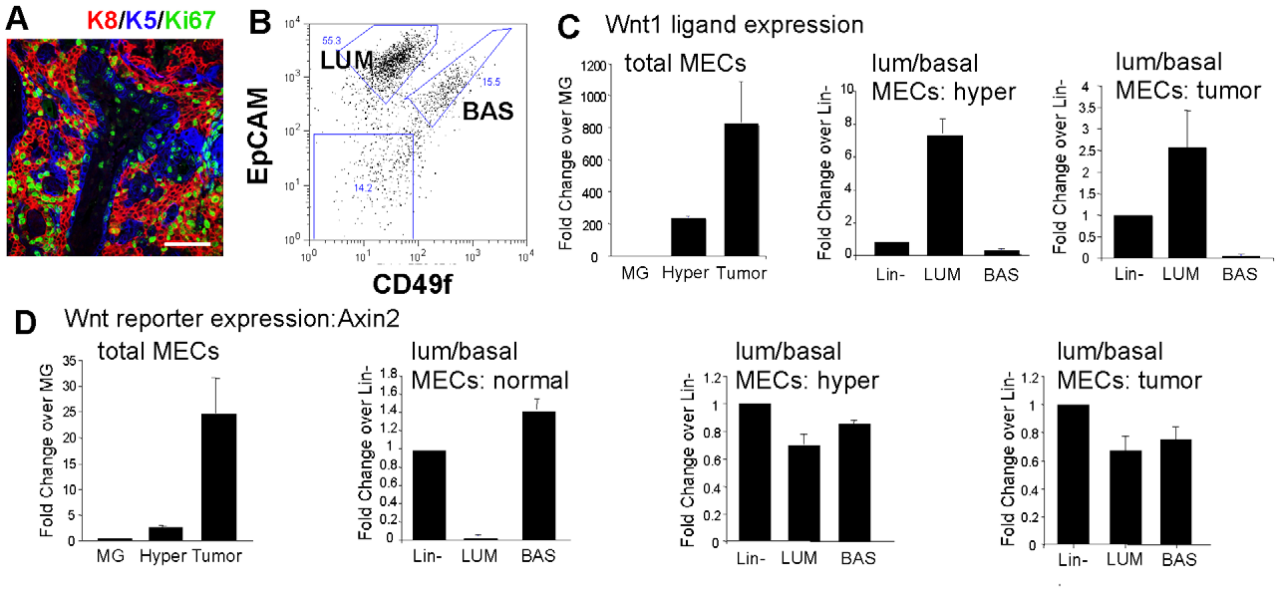
Despite lineage-specific expression of Wnt1 by purified basal and luminal cell subpopulations, both cell types express Axin2, and both serve as tumor initiating cells. (A) A representative MMTV-Wnt-1-induced tumor paraffin-embedded section stained with lineage-specific markers, keratin-8 (K8; luminal; red) and keratin-5 (K5; basal; blue), together with Ki67, a marker of cycling cells (green). Scale bar = 50 µm. (B) Flow cytometric separation of luminal and basal cells from an MMTV-Wnt1 induced tumor, based on their expression of EpCAM and CD49f (results are similar for at least 3 primary tumors; a representative gating tree is shown in Fig. S1). The purity of these fractions is shown in Fig. S3. (C, D) mRNA extracted from purified cell fractions was analyzed for relative expression of Wnt1 and the Wnt reporter, Axin2, by quantitative RT-PCR analysis (n = 2, triplicates). MG, mammary gland; Hyper, Wnt1-induced hyperplastic glands; BAS, basal cells; LUM, luminal cells; Lin−, CD45−, CD31− (lineage-negative) live cells (total un-separated cells, run through the cell sorter).

Wnt1-induced tumors are known to comprise cell variants related to the two mammary lineages, basal and luminal [9], [10], illustrated in Fig. 1A (stained with lineage-specific keratin markers, luminal marker K8 and basal marker K5). We used the relative expression of the cell surface molecules, EpCAM and CD49f (α6 integrin), to separate these cell types from Wnt1-induced mammary glands (Fig. S1), either the pre-neoplastic (so-called hyperplastic; hyper) glands or the tumors that develop later (shown in Fig. 1B and S2). Luminal cells were EpCAM^+^/CD49f^low^ and basal cells were EpCAM^+^/CD49f^high^. The proportion of basal cells increased in Wnt1-induced hyperplastic populations, as observed before using a modified cell separation protocol (Fig. S2 [10]). Staining of cells after sorting showed that these fractions were substantially pure; 99% of luminal cells expressed K8 (4% co-expressed K5) and the basal cell fraction was ≥95% K5+K8-negative (Fig. S3).

### Expression patterns of Wnt1 ligand and the Wnt reporter, axin2

To investigate the molecular expression of the receptor-ligand pair that comprise the essential paracrine signaling pathway sustaining these Wnt1-induced cell populations, we assayed mRNA expression for Wnt1 ligand, Lrp5 receptor and axin2, a Wnt reporter, in the separated cell types. Wnt1 ligand is not endogenously expressed, but was highly expressed in transgenic MECs, and super-induced in Wnt1 tumors (Fig. 1C). The MMTV-LTR directs expression to the luminal cells [20], [21]. The Wnt reporter, axin2, showed a pattern of induction that corresponded to the Wnt1 ligand expression in total MEC populations (Fig. 1C, D). In normal tissues, axin2 mRNA expression was restricted to basal cells. However, in luminal and basal cells extracted from Wnt1-induced MECs, axin2 mRNA was as highly expressed in luminal cells as basal cells.

### Basal tumor cells are enriched in tumor initiating cells, but luminal tumor cells can also serve as tumor stem cells

Interestingly, when we tested the tumor initiating cell activity of purified basal and luminal cells, the appearance of tumor stem cell activity correlated with the expression of Wnt reporter activity. Thus the frequency of tumor initiating cells (TICs) was approximately 1/1000 of the total tumor cell population. Though TIC activity was highly enriched in basal cells (10×), and relatively depleted in luminal cells (0.6×), the fact that luminal cells comprise the majority (61%) of the tumor cell population meant that approximately half the total TIC activity co-purified with luminal cells (Table 1). This led us to propose that the appearance of Wnt signaling activity in luminal cells was linked to their functional activation as tumor stem cells.

**Table 1:** Assay of Tumor Initiating Cell (TIC) frequencies for basal and luminal cell subpopulations from Wnt-induced tumors.

Cell fraction	% cells infraction	# of cells transferred	Take rate	Frequency of TICs (95% CI) (*p^GOF^*)	TICs per 10^6^ total cells (Lin−)	Fold enrichment
**Total (Lin−)**	100	4000	4/4	**1/935**	1070	1
		1000	3/4	(1/390–1/2240) (*0.39*)		
		500	1/4			
**Luminal**	60.8	4000	4/4	1/1506*	404	0.6
		2000	3/4	(1/697–1/3260) (*0.25*)		
		600	1/4			
		200	0/4			
**Basal**	4.3	200	4/4	1/87**	480	10.7
		100	2/4	(1/38–1/199) (*0.81*)		
		20	1/4			

Cell fractions purified by flow cytometry were assayed by isograft to mammary fat pads at limiting dilutions. We assume that if the significance of the goodness of fit calculation is >0.05, TIC frequencies can be calculated from limiting cell dilutions (the *p* ^GOF^ are shown below the TIC frequency; the goodness of fit calculation is done according to the limdil software, see Methods). Pairwise comparison of the sub-fractions of cells shows that the TIC frequency in the luminal and basal cells is significantly different (* p = 1.06×10^−5^), as was the TIC frequency for basal and total (Lin−) cells (** p = 2.7×10^−4^). To determine the absolute number of TICs in each fraction, the number of TICs per million **total** cells was calculated as the product of the number of cells in that subfraction, and the calculated TIC frequency. Fold enrichment is shown as the frequency of TICs in basal or luminal cells/frequency of TICs in the total population.

### Lrp5 protein becomes expressed in Wnt1-expressing luminal tumor cells, explaining the expression of the Wnt reporter

**Figure 2:**
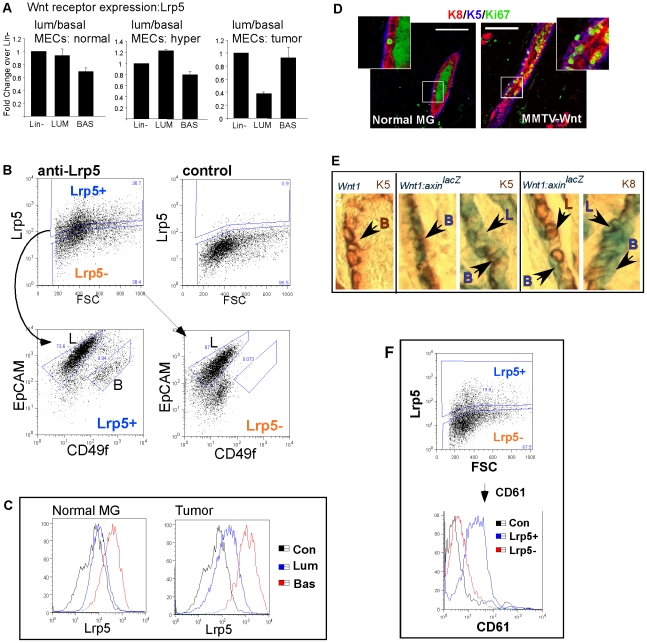
Lrp5 expression (acquired post-transcriptionally) correlates with the appearance of Axin2 in luminal cells. (A) mRNA extracted from purified cell fractions was analyzed for relative expression of Lrp5 by quantitative RT-PCR analysis as described for Fig. 1. (Lrp6 mRNA expression is broadly similar to Lrp5, shown in Fig. S4) (B) Tumor cells were incubated with anti-Lrp5 antibody, and analyzed by flow cytometry, using an isotype-matched negative control to define Lrp5+ cells. These samples were also stained with EpCAM and CD49f antibodies, and the cell surface phenotypes combined to show the luminal (L) or basal (B) cell identity of Lrp5+ and Lrp5− cells. (C) Another example of this assay is shown in histogram form, to reveal the overall level of expression for Lrp5 in luminal and basal cells in normal and tumor cells. (D) Paraffin sections from normal and Wnt-induced mammary glands were stained to illustrate the relative rates of division (green, Ki67) of basal (blue, K5) and luminal (red, K8) cells. Note that the green color stain in the lumens is a common artifact associated with non-specific binding to sticky luminal proteins. Scale bar = 50 µm. (E) *Axin2^lacZ^* [MMTV-Wnt1] (and control Wnt1) glands were stained in whole mounts for the *lacZ* reporter, followed by embedding, sectioning and immunocytochemical assay of basal (K5^+^) and luminal (K8^+^) cells. The panel on the LHS shows there was no background when the *Axin2^lacZ^* reporter was not present. The samples were incubated in x-gal substrate (B, basal; L, luminal; as indicated). The pattern of staining of *Axin^lacZ^* was heterogeneous, in some areas, stain was basal-specific, in others, there was also light staining throughout the luminal population, in others, focalized clusters of luminal cells showed high expression. (F) Lrp5+ cells were assayed for their expression of CD61, because CD61+ cells have been shown to be enriched for TIC activity in this tumor model (Vaillant et al 2008). These markers showed high co-expression.

Knowing that Lrp5 is singularly responsible for Wnt1-mediated effects in mammary glands [15], we evaluated the expression of Lrp5 in luminal and basal cells, to find out whether we could explain the appearance of axin2 mRNA expression. Analysis using qPCR showed that Lrp5 mRNA was expressed by both basal and luminal cells (Fig. 2A) in normal and Wnt1-induced MEC populations. Joshi et al. [23] have also observed the expression of Lrp5 mRNA in both basal and luminal cells (from normal glands, separated using a different protocol). This fact surprised us, given that we have previously observed a highly basal cell-specific pattern of Lrp5 protein expression in normal MECs (Fig. 2C and [16]). From this, we conclude that expression of Lrp5 protein is restricted to cells in the basal lineage based on a post-transcriptional mechanism. (Lrp6 showed a similar trend; cell surface Lrp6 protein was specific to basal cells, whilst the mRNA was expressed in both luminal and basal cells; Fig. S4). To evaluate whether we could account for the expression of axin2, we tested basal and luminal cells for their expression of Lrp5 by flow cytometry (Fig. 2B). All Lrp5-negative cells are luminal, but unlike normal MECs, some Wnt1-induced luminal cells were Lrp5-positive, though the relative amount of cell surface Lrp5 was 10× lower than basal cells (Fig. 2C). To find out which cells responded to the mitogenic Wnt1 signal, we evaluated the mitotic index of luminal and basal cells in Wnt1-induced mammary glands (Fig. 2D; compared to normal adult virgin glands that are not known to be Wnt-induced). Both basal and luminal cells were induced to divide, consistent with the idea that the mitogenic signal might be perceived by both cell types. To corroborate the qPCR results for axin2 expression in the luminal and basal cell populations defined by flow cytometry, we evaluated axin2 expression *in situ*, using the relative location of axin2-positive cells (together with subtype-specific counter-stains). Thus an *Axin2^lacZ^* knock-in reporter strain (see Methods) was crossed to MMTV-Wnt1 mice, and samples stained with K5 and K8 (Fig. 2E). As reported in other studies [21], [22], basal cells showed high expression of Axin2*^lacZ^* in Wnt1-induced hyperplastic glands. We found that luminal cells could also be positive, and that the pattern of staining was heterogeneous, including many lightly staining cells, and some focalized areas of dark staining (Fig. 2E). We conclude that luminal cells show low but significant Lrp5 protein expression, which is reflected in the expression of the axin2^lacZki^ allele in luminal cells. Note that the amount of endogenous axin2 mRNA was approximately equal in luminal and basal cells (Fig. 1D), whereas the expression of the axin2^lacZki^ allele appeared to be lower in luminal cells. This might be because whole mount samples are incubated with x-gal, and the substrate diffuses through the outer layer of basal cells before being available to the luminal cells (this problem of x-gal “exhaustion” was also described by Baker et al. [22]). Alternatively it might be because the stability of the lacZ protein in basal and luminal cell context may be different.

### Lrp5-positive cells account for the tumor initiating activity

If Lrp5 expression is required to close a paracrine Wnt1-signaling loop that determines cell survival and tumor initiating activity, then Lrp5-positive cells should include most of the tumor stem cells. Indeed, Lrp5-positive cells contained more than 90% of TIC activity (Table 2). A previous study showed that the majority of tumor initiating cell activity for tumors arising in this mouse model were CD61-positive [11]; we found that Lrp5-positive cells also express CD61 (Fig. 2F and S5), so these two cell surface markers are co-expressed. This suggests that the aberrant expression of Lrp5 in luminal cells also could apply to CD61 (and probably other proteins), and identified cells that had acquired stem cell function.

**Table 2:** Assay of TIC frequencies for Lrp5^+^ and Lrp^−^ cell subpopulations from Wnt-induced tumors.

Cell fraction	% cells in fraction	# of cells transferred	Take rate	Frequency of TICs (95% CI) (*p^GOF^*)	TICs per 10^6^ total cells (Lin−)	Fold enrichment
**Lrp5+**	36.7	2000	4/4	1/386*	951	2.4
		1000	2/2	(1/178–1/838) (*0.87*)		
		500	4/6			
		100	1/4			
**Lrp5−**	38.4	3000	3/4	1/4426*	87	0.2
		2000	1/4	(1/1862–1/10530) (*0.106*)		
		1500	1/2			
		1000	0/4			
		500	0/4			

Lrp5^+^ and Lrp5^−^ (Lin−) cell fractions were purified by flow cytometry, assayed by isograft to mammary fat pads at limiting dilutions, and results were reported as for Table 1. Pairwise comparison of the Lrp+ and Lrp− cells shows that the TIC frequency is significantly different (* p = 2.34×10^−5^).

### Luminal tumor stem cells regenerate tumors with a similar basal subpopulation

**Figure 3:**
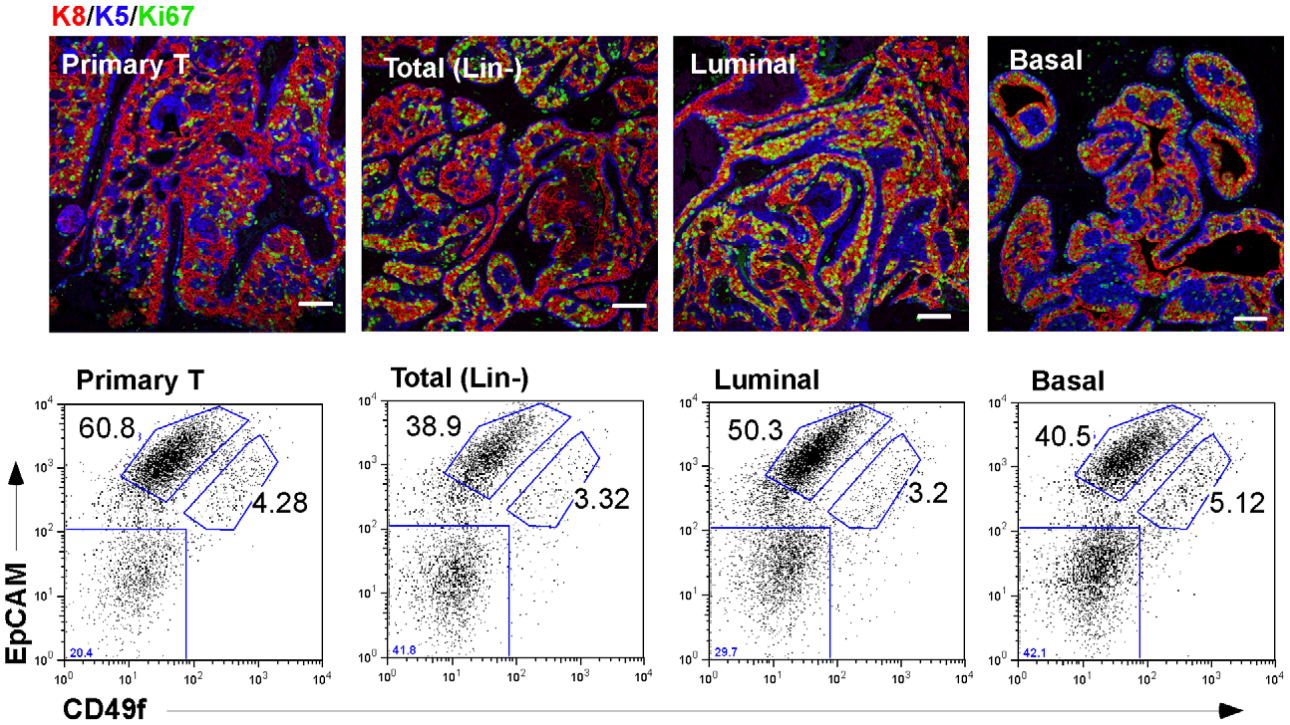
Functional evaluation of Wnt1-induced basal and luminal populations in vivo. Tumors regenerated from limiting dilutions of different cell fractions (Lin− total population, luminal or basal cells) were compared with samples of parental tumors immunostained for constituent cell types with K5 (blue), K8 (red), and Ki67 (green). Scale bar = 50 µm. These tumors were also analyzed by flow cytometry (using EpCAM and CD49f expression) to confirm the relative fate allocation of tumor subpopulations.

No matter what type of cell was used at limiting dilution to initiate tumor growth, tumors grew back at the same speed (3–4 weeks, data not shown) and recapitulated the primary tumor phenotype (Fig. S6). This robust pattern of intermingled differentiated cells is consistent with our suggestion that these cells are functionally inter-dependent. Not only that, but luminal cells regenerated tumors that comprise the same proportion of basal cells (Fig. 3), both by immunofluorescent assay and flow cytometry.

### Plasticity of cell differentiation in Wnt1-induced luminal cells

**Figure 4:**
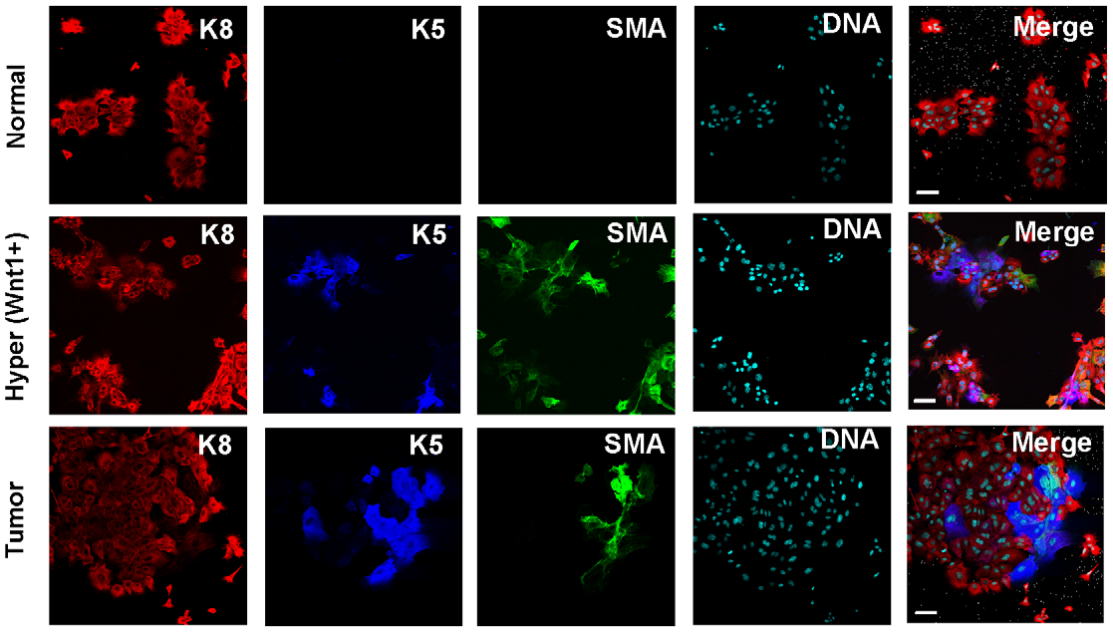
Functional evaluation of Wnt1-induced luminal cells in culture. To evaluate the differentiation potential of luminal cells from Wnt1-induced mammary glands, flow sorted luminal cells were placed into culture, and stained 4 days later for markers of cell fate (K5, SMA and K8), together with a nuclear counter-stain. Under these conditions, purified luminal cells (<1% K5-positive) from normal glands continue to express K8, and were 98.9% K5-negative after 4 days. However, purified luminal cells from MMTV-Wnt1 hyperplastic glands (also <1% K5-positive) show mixed fates after transfer to tissue culture, including 24% K5-positive cells. Similarly, cultures of purified luminal cells from MMTV-Wnt1-induced tumors develop a similar proportion (21%) of K5-positive cells (n = 2, triplicates). Note that some cells are K5-positive, some are SMA-positive and most are positive for both. For BALB/c MECs, this is typical of the basal/myoepithelial lineage (*in vivo* and directly after isolation), and probably reflects an evolution of phenotype during differentiation (cells expressing SMA alone are most differentiated; data not shown). The corresponding cultures of basal cells from all three types of MEC population are shown in Fig. S7. Scale bar = 50 µm.

In the simplest scenario, the acquisition of Lrp5 by luminal cells might be expected to result in monotypic luminal-type tumors upon transplantation. The fact that basal cells re-appear suggests instead that the post-transcriptional induction of Lrp5 may not be sufficient to maintain robust tumor growth. Consistent with this, we observed that purified Wnt1-expressing luminal cells did not show the pattern of mono-lineal expansion *in vitro* that is associated with normal cells (Fig. 4). Instead, Wnt1-induced luminal cells “retro-differentiated” to basal cells (reversing the usual basal-luminal pattern of differentiation). We propose that this same pattern of growth exists *in vivo*, to account for the appearance of basal cells in tumors initiated from luminal cell antecedents.

## Discussion

**Figure 5:**
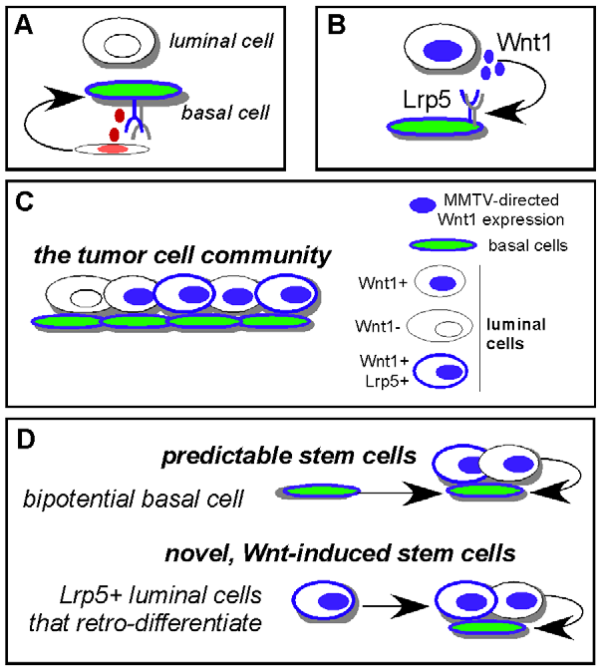
Summary diagram. (A) In normal mammary epithelium, the Wnt source that maintains Lrp5+ mammary stem cells is indicated by a red-colored nucleus in a non-epithelial cell type, inferred from [40]. (B) For Wnt1-induced mammary tumors, we have illustrated a paracrine signaling network that depends upon interactive basal and luminal cells. Wnt1 ligand and Lrp5 receptor are necessary and sufficient to maintain tumors (Lindvall et al., 2006). Wnt1, under the control of the MMTV-LTR sequence, is expressed by luminal cells (this cis sequence is only upregulated in luminal cells). Lrp5 protein is typically expressed by basal cells. (C) Cells that comprise the tumor cell community include (Lrp5+) basal cells that can differentiate to luminal cells to provide a ligand-expressing luminal partner. We have shown that some of these luminal cells also express Lrp5. (D) All cells with stem cell activity express Lrp5 and are able to regenerate robust mixtures of basal and luminal cells that support tumor growth by paracrine interactions. Thus, basal cells are predictable stem cells, since they differentiate to Wnt1-expressing luminal cells, whereas luminal cells are novel, Wnt-induced stem cells that express sufficient Lrp5 to enable their survival, but also show fate plasticity, to enable retro-differentiation to basal cells.

Using a well-established mouse basaloid breast tumor model, we show that two differentiated cell types that comprise this tumor are interactive. Thus, the differentiation of luminal and basal mammary epithelial cells from a common progenitor is associated with the expression of oncogenic Wnt1 ligand by the luminal cells (under the control of the MMTV-LTR), and the expression of its cognate functional receptor, Lrp5, predominantly on the surfaces of the basal cells (see scheme presented in Fig. 5B). Thus differentiation, or (more broadly) fate specification, is required to maintain the expression of the oncogenic signaling pathway, and is likely to drive the retention of both cell types in this basaloid tumor type. In the tumors we describe, there are more luminal cells than basal cells, and this proportion is robust and constant (upon transplantation). Similar selective pressures could operate to maintain the corresponding cell types in human basaloid breast tumors (and presumably in other tumor types that show evident heterotypic differentiation), and these are likely to exploit the interactive paracrine reactions that usually control development and morphogenesis. Indeed, recent data extracted from transcriptional arrays of basal and luminal cells show reciprocal expression of several ligand-receptor pairs [23], [24]. Furthermore, the heterogeneous cell populations that comprise several cultured breast cancer cell lines have been shown to be inter-dependent, and survive via a paracrine CXCR1/IL8 pair, the receptor expressed on the ALDH+ tumor initiating cell compartment and the ligand by the tumor bulk cells [25]. Quite analogously, recent data has shown that estrogen-treatment of MCF cell cultures induces the expression of Fgf by the ERα-positive majority, maintaining an FGFR/Tbx3 positive tumor initiating cell minority [26].

For a tumor that depends upon cooperation between two cell types, we predict that any cell that can differentiate to generate both types of cell (to close the mitogenic loop) will have tumor initiating activity *in vivo*. If we compare these Wnt1-induced basaloid tumors with basaloid tumors induced by carcinogen administration (Kim, Roopra and Alexander, submitted), they look remarkably similar (by histopathology, flow cytometric immunophenotypes, and relative proportion of basal and luminal cells). However, in carcinogen-induced tumors, only basal tumor cells have stem cell activity. This is predictable, since it recapitulates the normal bi-potential activity of the basal cell compartment. In contrast, Wnt1-induced tumors contain novel luminal stem cells, and the expression of Lrp5 protein by these cells supports their exceptional stem cell activity (see scheme of Fig. 5C, D). Though acquisition of Lrp5 expression by luminal cells (together with the ability to express Wnt1, to close the essential loop) might be predicted to be sufficient to drive the formation of homotypic, luminal tumors, this is not what we observed *in vivo*. Instead, tumors initiated by luminal cells comprise the same mixture of cell types as tumors initiated by basal cells. This might be due to the molecular mechanism underlying this phenomenon; thus Wnt1 expression induces the cell surface presentation of Lrp5 (at low but functionally significant amounts) from an mRNA not usually translated in luminal cells. This change may not be sufficiently durable to maintain tumor growth. It is becoming increasingly evident that the regulation of translation of key mRNA species governs differentiation; for example, a group of mRNAs (comprising 2% of total transcripts) was shown to be specifically translated during differentiation of embryonic stem cells, including Wnt1 [27]. One obvious mechanism that could explain selective translation is the lineage-specific expression of miRNAs, already known to regulate growth and differentiation of mammary gland [28].

To maintain tumor growth, we propose that a second property of Wnt-induced luminal cells is important. We demonstrated that unlike the usually lineage-committed luminal cells from normal glands, purified Wnt1-expressing luminal cells differentiate spontaneously to basal cells in culture. In this context, “luminal cells” are defined as cells that co-purify according to their flow cytometric profile (EpCAM^hi^, CD49f^med^). (Note that 4% of Wnt1-induced luminal cells co-express cytokeratins 5 and 8; this may be a marker of plasticity/stem-ness). We propose that plasticity of luminal cell phenotype in the presence of Wnt1 could explain why tumors can be reconstituted from purified luminal cell fractions, because it allows the optimal accumulation of basal cells to promote population growth. These observations propose a distinct and novel function for Wnt signaling during oncogenesis, as a collaborator for paracrine cell pairs, when plasticity will enable their efficient differentiation.

Many solid tumors have been analyzed for the presence of tumor stem cells by prospective flow cytometric analysis, using relatively arbitrary cell surface markers. Though this has been successful, the discrimination of tumor initiating cells into one fraction or another tends to be a matter of chance. On the other hand, if a cell surface marker that is functionally important to stem-ness can be applied, this process could become rationalized. In this study, we show that tumor-initiating cells are enriched in the Lrp5-positive fraction, regardless of other phenotypes. However, we do not claim this as a general breast tumor stem cell marker, but only when Wnt signaling is necessary and sufficient for tumor growth. In general terms, expression of the receptor of an oncogenic paracrine signaling pathway is likely to mark the tumor stem cell community.

The Wnt1-induced mouse mammary model is significant to our understanding of tumor stem cells, for at least two reasons. Firstly, since the preneoplastic mammary gland was shown to accumulate mammary stem cells, it has been generally assumed that stem cells dominate tumor initiation [9], [10]. Secondly, tumors arising in this model comprise basal and luminal cell equivalents, a feature also characteristic of basaloid human breast tumors [5], [6], [29]. Note that we make no claim that the [MMTV-Wnt1] model described in this manuscript is an accurate one for basaloid tumors. Although several Wnt ligands and inhibitors are expressed in normal breast and breast tumors, the functional significance of Wnt signaling in basaloid (or indeed luminal) breast tumors has not yet been directly tested. However, in support of Wnt signaling as an etiology for breast cancer is the oncogenicity of ectopic Wnt signaling in mouse models [10], [30], functional data from basaloid breast cancer cell lines (for example, [31], and reports of heterogeneous nuclear staining of β-catenin (which may indicate active Wnt signaling) in tumors in vivo [32].

Models of this aggressive tumor type are especially valuable since little is understood about their heterogeneous molecular and cellular etiology. Recently, basaloid human tumors from BRCA1-mutant women provided evidence for a different type of tumor stem cell, derived from a luminal cell-of-origin [33], [34]. Similarly, p53 deficiency produces aggressive basaloid tumors in mice [8], [35]. For both of these tumor types, it is not clear whether there are two distinct and cell fate-specified subpopulations. It may instead be true that these tumor cells co-express growth-promoting pathways that are usually separated between basal and luminal progenitors. Indeed, when p53 null alleles are crossed into the [MMTV-Wnt1] transgenic model, tumors arise earlier and show little evidence of differentiation [36].

Wnt1-induced tumors require continuous expression of Wnt1 [13]. Prior analysis of these tumors using functional assays of tumor initiating potential have showed that the tumor-generating cells had a predominantly basal character, and they were enriched in a Thy1+/CD24+ cell sub-population (1% of total Lin− cells, showing 35× enrichment of functional activity) [12]. Another study observed that stem cells and tumor initiating activity could be retrieved from Wnt-induced mammary epithelial cell populations using the cell surface marker CD61 (β3 integrin) [11]. In this study, stem cell activity retrieved from a luminal-enriched subpopulation constituted less than 10% of total. Note that only our study has systematically reported the basal/luminal fate allocation of separate populations isolated by flow cytometry, so it is difficult to directly compare data sets.

Our results lead us to these conclusions: (1) By comparing the proteome of constituent cell types of a heterogeneous tumor, it should be possible to list candidate paracrine receptor-ligand pairs that could be responsible for maintaining the tumor. This could provide a discovery mechanism for highly druggable pathways. (2) It will be important to know which cells show plasticity, since these are key chemotherapeutic targets to produce durable chemotherapeutic responses.

## Materials and Methods

### Ethics Statement

All experiments that included the use of mice were approved by the University of Wisconsin IACUC (protocol number MO1422; Institutional assurance number for University of Wisconsin-Madison A-3368-01). For the studies we describe, there are no suitable alternative approaches, and care is taken to minimize animal distress.

### Mouse Strains and Tumor Dissociation

The source and characteristics of MMTV-Wnt1 (C57BL/6) mice have been described previously [37]. Wnt reporter mice with a heterozygous Conductin^+/lz^ lacZ allele (Axin2^lacZki^) were provided by Walter Birchmeier [38], and we thank R. Nusse (Stanford) for their distribution. The Institutional Animal Care and Use Committee at the University of Wisconsin-Madison approved all experimental protocols. Primary mammary epithelial cell suspensions from wild-type or transgenic mice were prepared as described [16]. Tumors from transgenic mice were dissociated as described in Kim, Roopra and Alexander (submitted).

### Cell Sorting and Transplantation

Prior to flow cytometry, dissociated cells were stained with the following antibodies: APC-conjugated anti-CD45 (Cat.# 559864; clone number 30-F11; 1 µg/ml), APC-conjugated rat anti-mouse CD31 (Cat.# 551262; clone number MEC13.3; 1 µg/ml), FITC-conjugated CD49f (Cat.# 555735; clone number GoH3; 30 µl/ml) all from BD Biosciences, and PE-conjugated EpCAM (Cat.# 118206; clone number G8.8; 0.5 µg/ml) and FITC-conjugated CD61 (Cat.# 104305; 10 µg/ml) from BioLegend (San Diego, CA). To overlay the expression of Lrp5 or CD61 onto the EpCAM / CD49f profile, cells were pre-stained with CD31/45, CD49f, and EpCAM as described (Kim, Roopra and Alexander submitted) and then 2 µl of Lrp5 (41–130 Ascites fluid, Cat.# H00004041-M01A AbNova, Taipei City, Taiwan) or 2 µl of biotin-conjugated anti-mouse/rat CD61 (Cat. # 13-0611-81; clone number 2C9.G3; 10 µg/ml; eBiosciences) was added (10^6^ cells in 100 µl) for another 30 mins/4°C. The cells were washed and incubated with anti-mouse IgG-Alexa 405 (Cat.# A31553) or streptavidin-conjugated Pacific Blue (Cat.# S11222) from Molecular Probes (Eugene, OR) for 30 minutes on ice, and analyzed on a FACS Vantage cell sorter equipped with DiVa software (Becton Dickson, Franklin Lakes, NJ). Typical gating trees for tumor cell populations are shown in Fig. S1. Populations of tumor cells sorted by flow cytometry were transplanted at limiting dilutions, and tumor initiating cell frequency was calculated using limdil software (http://bioinf.wehi.edu.au/software/limdil). Results are typically displayed as dot plots for cells isolated from single tumors (results were replicated at least twice), or for pooled mammary epithelial cell preparations from ≥15 control mice or ≥5 MMTV-Wnt1 induced transgenic mice. This report follows the recommendations made by Alexander et al. [39].

### Immunofluorescent staining protocols

Methods for staining tumor tissue sections and sorted cells on slides were as described [16]. Primary antibodies used for immunofluorescence were: rabbit anti-keratin 5 (Covance, Madison, WI), rat anti-keratin 8 (Troma-I) (Developmental Studies Hybridoma Bank, University of Iowa), FITC conjugated mouse anti-α smooth muscle actin (Sigma, St. Louis, MO), and mouse anti-Ki67 (BD Biosciences). Secondary antibodies were: Pacific Blue goat anti-rabbit IgG, Alexa Fluor 546 goat anti-rabbit or rat IgG, and Alexa Fluor 488 goat anti-rat or mouse IgG from Molecular Probes (Eugene, OR). TO-PRO-3 (Molecular Probes) was used for nuclear DNA counterstaining and immunofluorescent stains were visualized on a confocal microscope (BioRad MRC1024).

### Immunoperoxidase and lacZ staining of paraffin sections from Axin2^lacZki^ mammary glands

Inguinal mammary glands were fixed in 4% paraformaldehyde for 1 h at room temperature and washed in lacZ wash buffer (0.01% sodium deoxycholate, 0.02% NP-40 and 0.02% IGEPAL in PBS) for 15 mins. Glands were compressed between microscope slides, incubated overnight in the wash buffer at 4°C to facilitate penetration of the staining solution, rinsed and stained (overnight) in X-gal staining solution (1 mg/ml 5-bromo-4-chloro-3-indolyl-β-D-galactopyranoside, 5 mM potassium ferrocyanide, 5 mM potassium ferricyanide, 2 mM MgCl_2_ in PBS). After paraffin embedding, sections were incubated with hydrogen peroxide to quench endogenous peroxidase activity, processed using citric acid antigen retrieval and stained using the primary antibodies described above, followed by biotinylated anti-rabbit or anti-rat IgG (Vector Laboratories, California), and the HRP/DAB detection kit (according to manufacturer's instructions, Abcam, Cambridge, MA).

### Primary culture of purified cell fractions

To culture primary epithelial cells, 8 well-chamber slides (Nalge Nunc International, Naperville, IL) were coated with Matrigel and sorted cells were plated at 10 000 cells per well in DMEM/F12 (Invitrogen, Carlsbad, CA) plus 2% FBS (Harlan Laboratories, Indianapolis, IN), 10 µg/ml insulin (Sigma), 100 U/ml Penicillin/Streptomycin (Invitrogen, Carlsbad, CA), and 20 ng/ml EGF (R&D System, Minneapolis, MN). Cells were cultured for 4 days and then stained with K8, SMA, and K5 as described above.

### Quantitative RT-PCR

Total RNA was collected using the PicoPure RNA Isolation Kit (Arcturus, Mountain View, CA) according to manufacturer instructions and processed as described [16]. Each sample was analyzed in duplicate with an ABI 7900-HT (Applied Biosystems, Foster City, CA) as follows: 1 cycle of 50°C/2 mins, 95°C/2 mins, followed by 40 cycles of 95°C/15 seconds, 55°C/30 seconds and 72°C/30 seconds, followed by a melt-curve analysis. The data were analyzed using the SDS2.2.2 software (Applied Biosystems) and the reference genes, YWHAZ and hypoxanthine-guanine phosphoribosyltransferase (HPRT) were used for normalization of data by the ΔΔ^Ct^ method. Primer sequences are listed in File S1 (sequences are listed 5′ to 3′; primers are designed to span intron-exon boundaries). Results reported describe triplicate technical analyses, representative of at least two separate experiments, as indicated.

## Supporting Information

Fig. S1 **Representative gating procedures for luminal and basal cells from MMTV-Wnt1 tumors.** Antibody staining and flow cytometric analyses were performed as described in Materials and Methods and Alexander et al. [39].
(DOCX)

Fig. S2 **Representative separations of luminal and basal cells from normal and MMTV-Wnt1 hyperplastic glands.** Comparison of flow cytometric profiles of basal and luminal cells from non-neoplastic (hyperplastic) populations, for comparison with Fig. 1B.
(DOCX)

Fig. S3 **Evaluation of purity of luminal and basal sub-populations of flow-sorted tumor cells.** (A) Cytosplats of cells purified by flow cytometry were evaluated for expression of luminal (K8) and basal (K5 and SMA) markers by immunocytochemistry (with a DNA counterstain to reveal total cell numbers). (B) Basal and luminal cell fractions were analyzed by qPCR for the relative expression of K5 and K8. Immunostaining and qPCR analysis of lineage-specific markers of basal and luminal cell sub-populations, to justify their separate analysis (throughout).
(DOCX)

Fig. S4 **Analysis of relative Lrp6 mRNA expression in basal and luminal sub-populations.** For comparison with Fig. 2A (Lrp5 mRNA expression), Lrp6 mRNA was analyzed by qPCR.
(DOCX)

Fig. S5 **Flow cytometric analysis of CD61 expression together with EpCAM/CD49f.** This analysis is for comparison with Fig. 3A, which shows the co-expression of CD61 and Lrp5. The two panels to the left show the gating procedure for CD61-positive and CD61-negative cells, followed by overlay onto the EpCAM/CD49f staining for resolution of the basal and luminal cell populations (identified as for Fig. 3A).
(DOCX)

Fig. S6 **Morphology of tumors regenerated from basal or luminal tumor initiating cells.** H&E stained paraffin sections of a representative primary tumor, and tumors regenerated from total Lin−, basal or luminal cell fractions. (These histological assays are for comparison with the samples stained for their expression of lineage specific markers shown in Fig. 3C).
(DOCX)

Fig. S7 **Differentiation of basal cell-derived cultures from normal and Wnt1-induced cell populations.** Basal cell cultures prepared according to the methods described for Fig. 3C (showing the differentiation of the luminal cell fractions from corresponding mice) were assayed for the expression of lineage-specific markers (K5 and SMA, basal cell markers; K8 luminal cell marker). For C57Bl6 mammary epithelial cells, the majority of basal cells express K5, but only some (co-)express SMA (our data suggests that SMA is a marker of terminally differentiated myoepithelial cells).
(DOCX)

File S1 Primer sequences for Quantitative RT-PCR.
(DOC)
