# Supplementary figures and images for: Differentiation Generates Paracrine Cell Pairs That Maintain Basaloid Mouse Mammary Tumors: Proof of Concept
Source: PLoS One. 2011 Apr 26;6(4):e19310. doi: 10.1371/journal.pone.0019310 (PMC3082567; doi:10.1371/journal.pone.0019310)

**Figure S1. Representative gating procedures for luminal and basal cells from MMTV-Wnt1 tumors.**


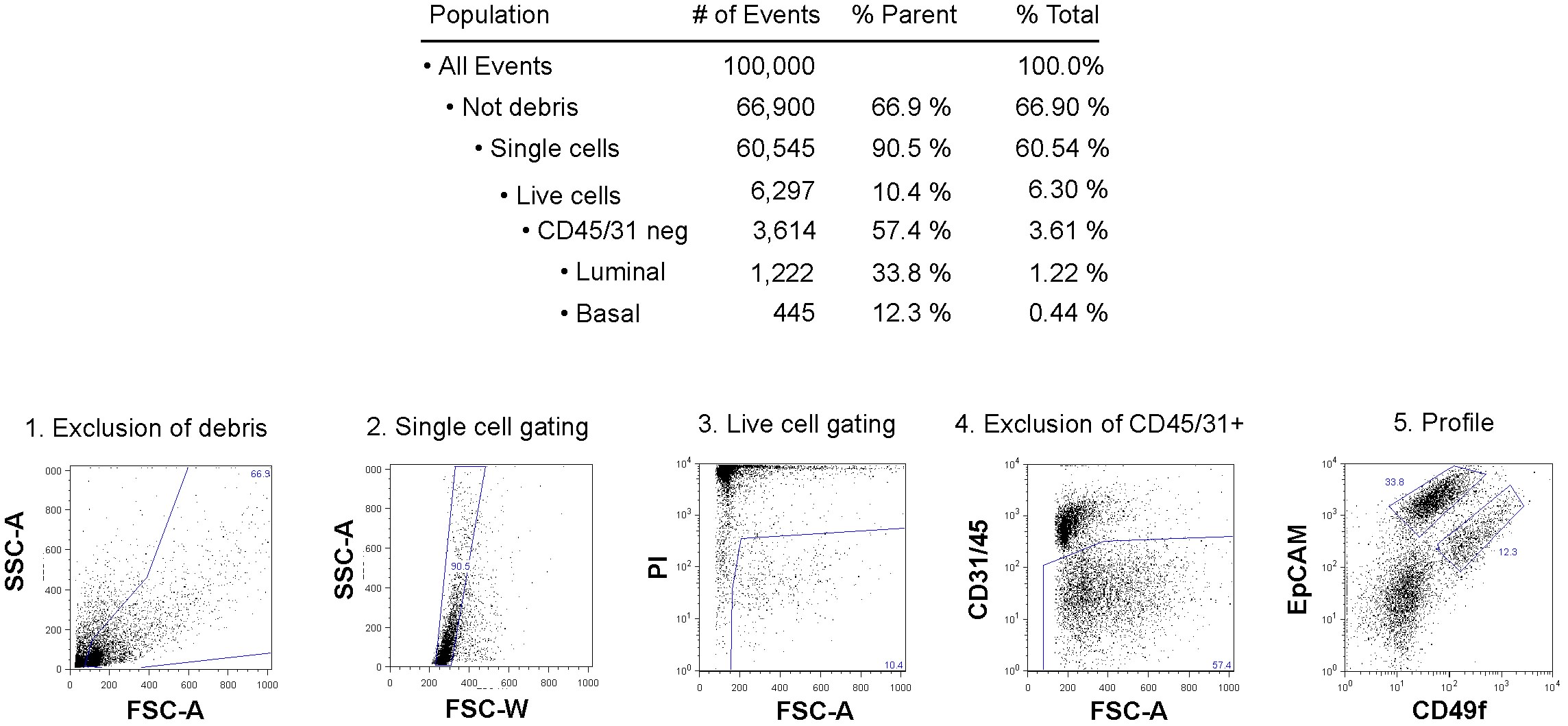

Supplement: Fig. S1 — Representative gating procedures for luminal and basal cells from MMTV-Wnt1 tumors. Antibody staining and flow cytometric analyses were performed as described in Materials and Methods and Alexander et al. [39]. (DOCX) [file pone.0019310.s001.docx]

**Figure S2. EpCAM / CD49f profiles of normal and Wnt1-induced hyperplastic populations**


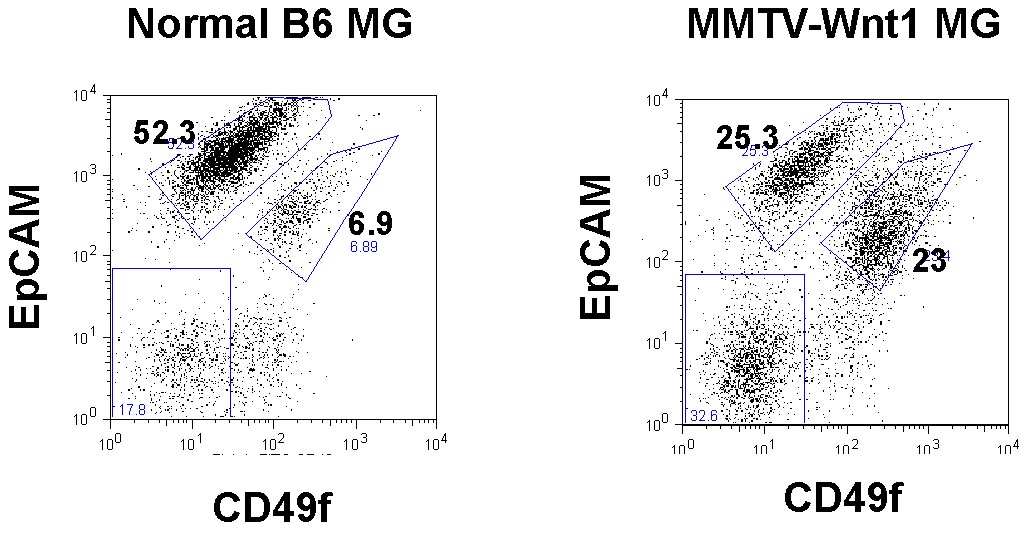

Supplement: Fig. S2 — Representative separations of luminal and basal cells from normal and MMTV-Wnt1 hyperplastic glands. Comparison of flow cytometric profiles of basal and luminal cells from non-neoplastic (hyperplastic) populations, for comparison with Fig. 1B. (DOCX) [file pone.0019310.s002.docx]

**Figure S3. Evaluation of purity of luminal and basal sub-populations of flow-sorted tumor cells**

**
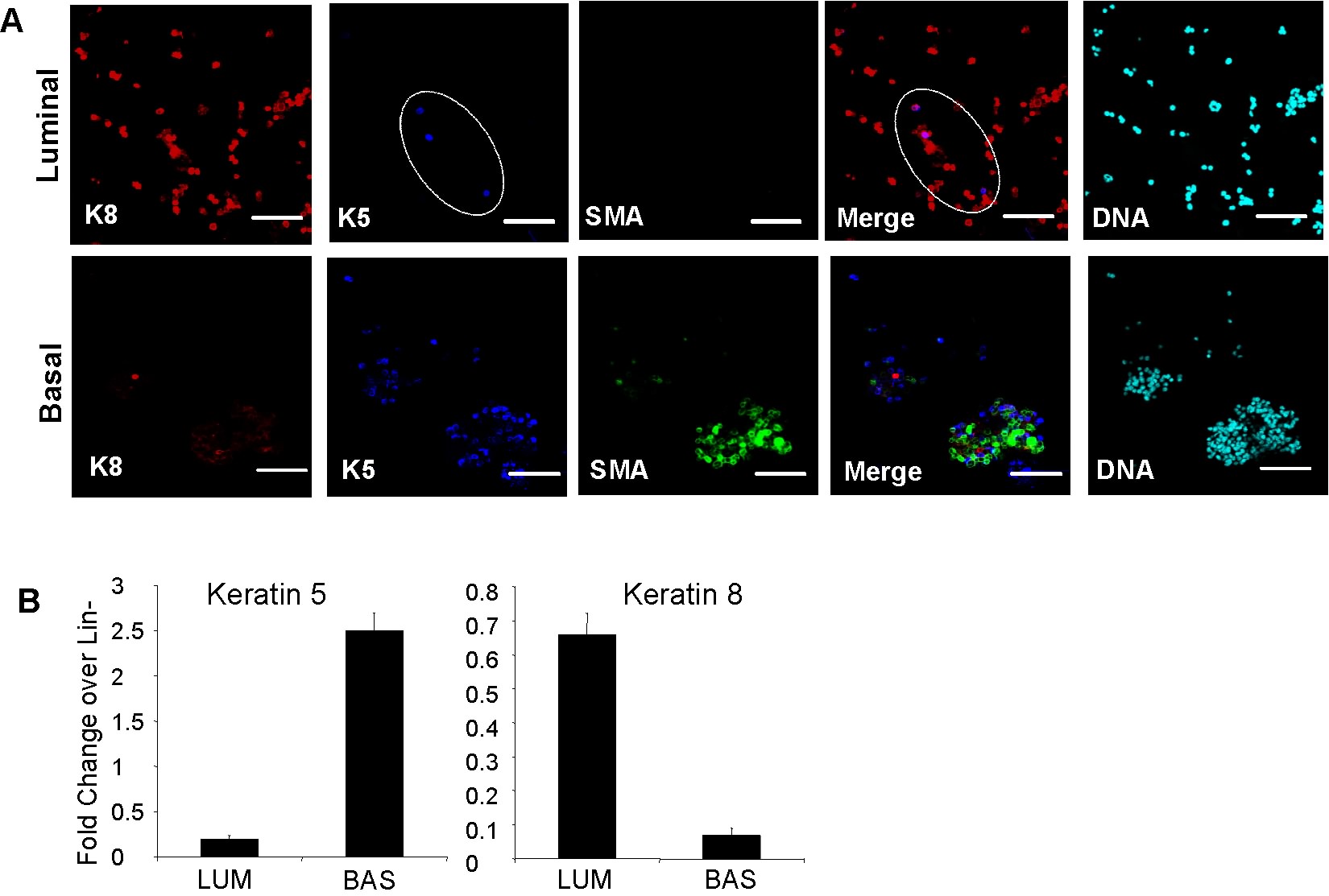
**

Supplement: Fig. S3 — Evaluation of purity of luminal and basal sub-populations of flow-sorted tumor cells. (A) Cytosplats of cells purified by flow cytometry were evaluated for expression of luminal (K8) and basal (K5 and SMA) markers by immunocytochemistry (with a DNA counterstain to reveal total cell numbers). (B) Basal and luminal cell fractions were analyzed by qPCR for the relative expression of K5 and K8. Immunostaining and qPCR analysis of lineage-specific markers of basal and luminal cell sub-populations, to justify their separate analysis (throughout). (DOCX) [file pone.0019310.s003.docx]

**Fig. S4. Analysis of relative Lrp6 mRNA expression in basal and luminal sub-populations**


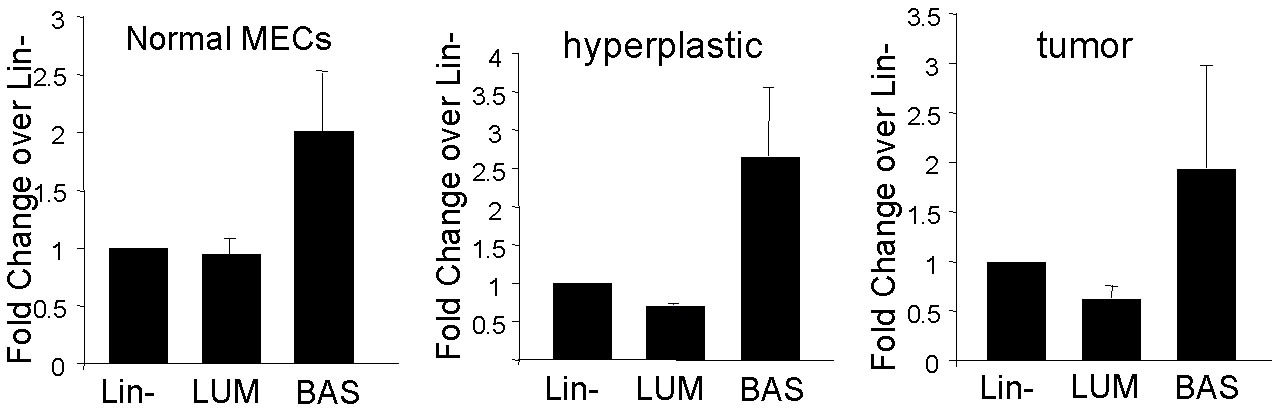

Supplement: Fig. S4 — Analysis of relative Lrp6 mRNA expression in basal and luminal sub-populations. For comparison with Fig. 2A (Lrp5 mRNA expression), Lrp6 mRNA was analyzed by qPCR. (DOCX) [file pone.0019310.s004.docx]

**Fig. S5. Overlay of CD61 expression onto the EpCAM/CD49f profile**.


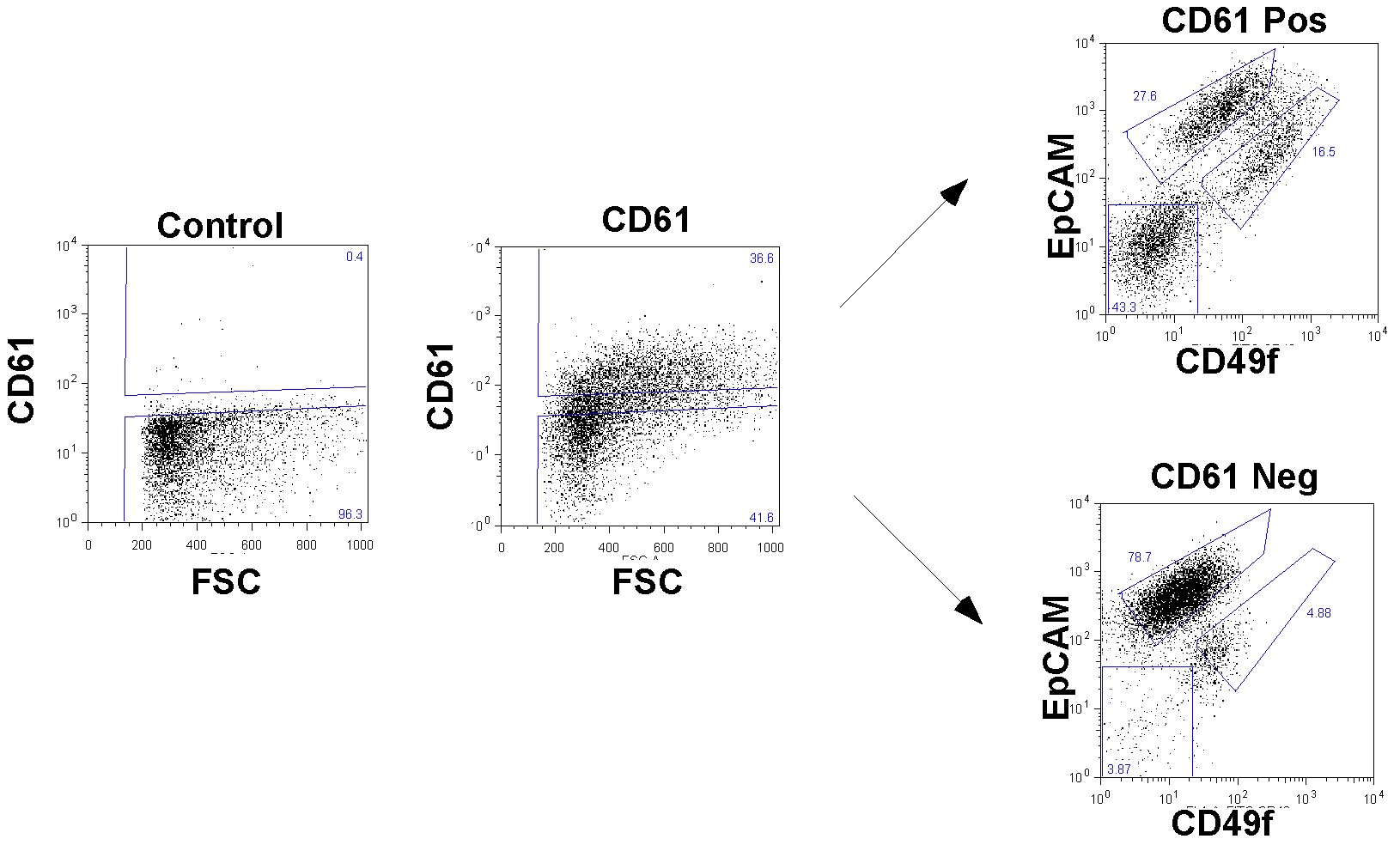

Supplement: Fig. S5 — Flow cytometric analysis of CD61 expression together with EpCAM/CD49f. This analysis is for comparison with Fig. 3A, which shows the co-expression of CD61 and Lrp5. The two panels to the left show the gating procedure for CD61-positive and CD61-negative cells, followed by overlay onto the EpCAM/CD49f staining for resolution of the basal and luminal cell populations (identified as for Fig. 3A). (DOCX) [file pone.0019310.s005.docx]

**Fig. S6. Morphology of tumors regenerated from basal or luminal tumor initiating cells.**


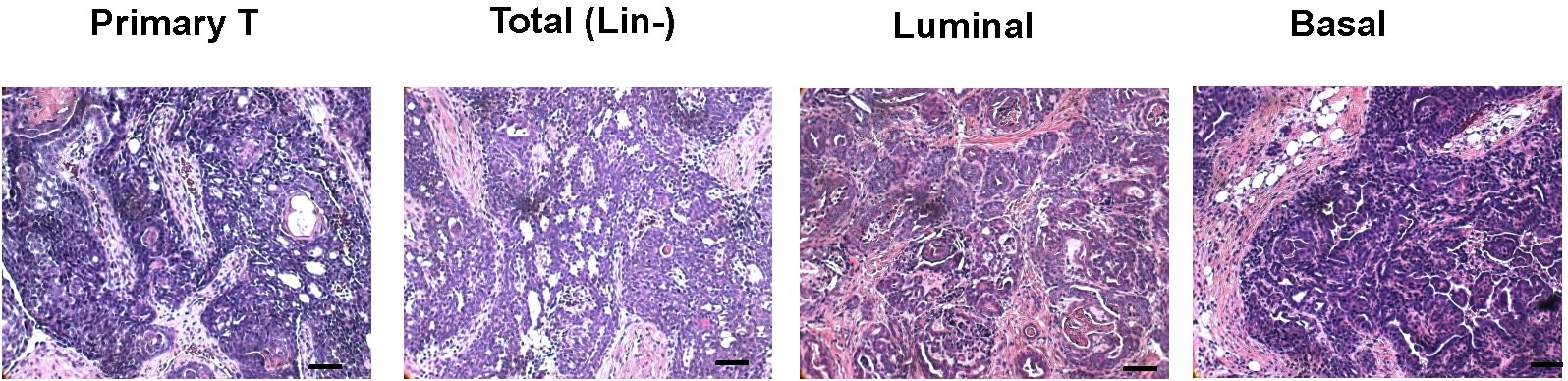

Supplement: Fig. S6 — Morphology of tumors regenerated from basal or luminal tumor initiating cells. H&E stained paraffin sections of a representative primary tumor, and tumors regenerated from total Lin−, basal or luminal cell fractions. (These histological assays are for comparison with the samples stained for their expression of lineage specific markers shown in Fig. 3C). (DOCX) [file pone.0019310.s006.docx]
