# Supplementary material for: Differentiation Generates Paracrine Cell Pairs That Maintain Basaloid Mouse Mammary Tumors: Proof of Concept
Source: PLoS One. 2011 Apr 26;6(4):e19310. doi: 10.1371/journal.pone.0019310 (PMC3082567; doi:10.1371/journal.pone.0019310)

**Fig. S7. Differentiation of basal cell-derived cultures from normal and Wnt1-induced cell populations.**


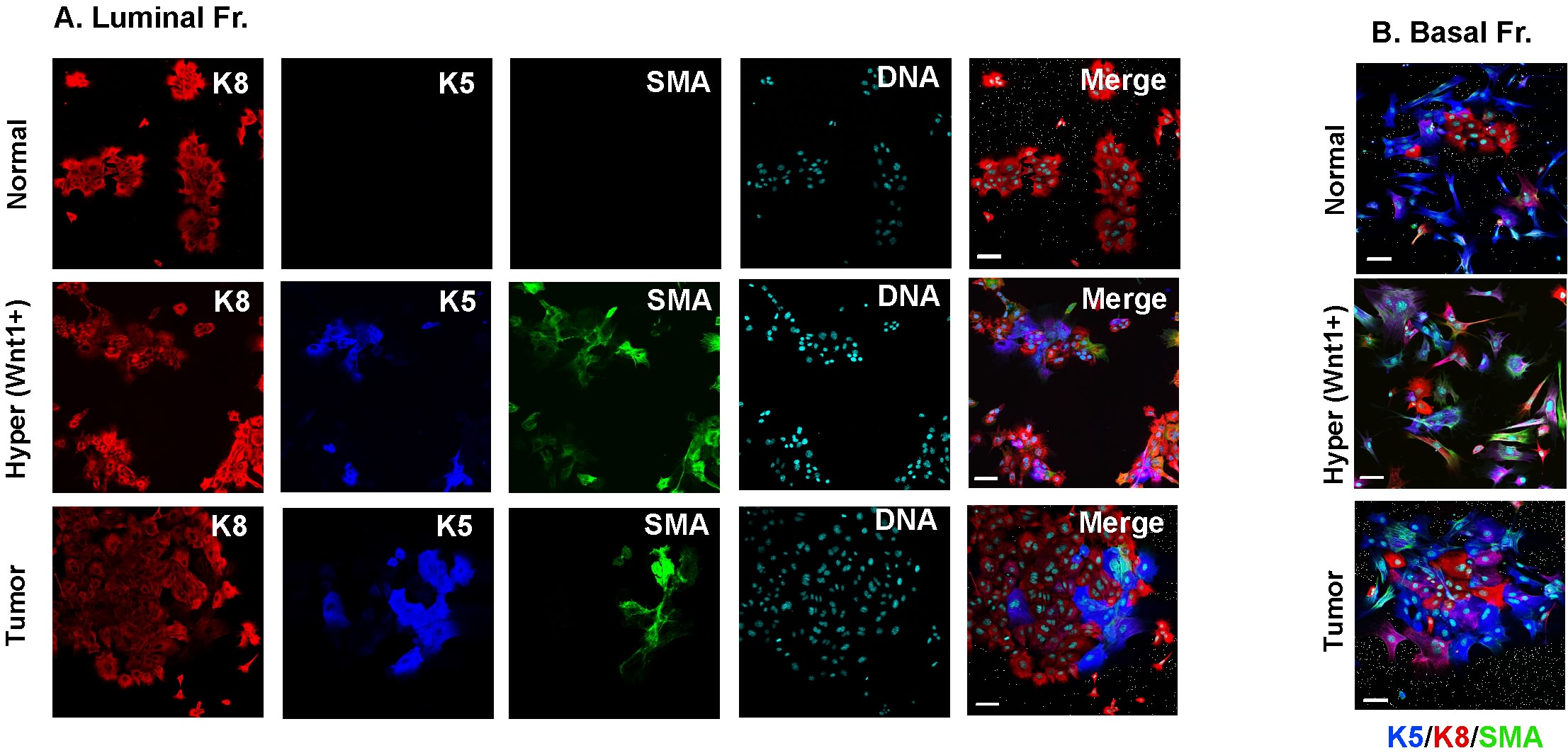

Supplement: Fig. S7 — Differentiation of basal cell-derived cultures from normal and Wnt1-induced cell populations. Basal cell cultures prepared according to the methods described for Fig. 3C (showing the differentiation of the luminal cell fractions from corresponding mice) were assayed for the expression of lineage-specific markers (K5 and SMA, basal cell markers; K8 luminal cell marker). For C57Bl6 mammary epithelial cells, the majority of basal cells express K5, but only some (co-)express SMA (our data suggests that SMA is a marker of terminally differentiated myoepithelial cells). (DOCX) [file pone.0019310.s007.docx]
