# Supplementary material for: Differentiation Generates Paracrine Cell Pairs That Maintain Basaloid Mouse Mammary Tumors: Proof of Concept
Source: PLoS One. 2011 Apr 26;6(4):e19310. doi: 10.1371/journal.pone.0019310 (PMC3082567; doi:10.1371/journal.pone.0019310)
Supplement: File S1 — Primer sequences for Quantitative RT-PCR. (DOC) [file pone.0019310.s008.doc]

**Materials and Methods**

Primer sequences are listed below (5’ to 3’; primers are designed to span intron-exon boundaries). Primers were obtained from Integrated DNA Technologies (IDT) and designed using the manufacturer’s software (<http://www.idtdna.com/analyzer/Applications/OligoAnalyzer>).

| **Primers** | **Sequences** | Position |
| --- | --- | --- |
| YWHAZ forward | AAG ACA GCA CGC TAA TAA TGC | Exon 6 |
| YWHAZ reverse | TTG GAA GGC CGG TTA ATT TTC | Exon 7 |
| HPRT forward | TTA TCA GAC TGA AGA GCT ACT GTA ATG | Exons 3 and 4 |
| HPRT reverse | TTA CCA GTG TCA ATT ATA TCT TCA ACA ATC | Exons 5 and 6 |
| Axin2 forward | TTT GGC ACA GCT AGA GGA AG | Exon 8 |
| Axin2 reverse | TGG CTC TTT GTG ATC TTC TGG | Exon 9 |
| Wnt1 forward | TCT TCG GCA AGA TCG TCA AC | Exon 2 |
| Wnt1 reverse | CAG GTG CAG GAC TCG ATG | Exon 3 |
| K5 forward | GTA CCA GGA GCT CAT GAA CAC | Exon 7 |
| K5 reverse | TTG ACT GGT CCA ACT CCT TC | Exon 8 |
| K8 forward | ATC GAG ATC ACC ACC TAC CG | Exon 7 |
| K8 reverse | TGA AGC CAG GGC TAG TGA | Exon 9 |
| Lrp5 forward | AAG CAA CAG TGT GAC TCC TTC |  |
| Lrp5 reverse | GGG AGA GGA TGA TAC CAA TGA C |  |
| Lrp6 forward | GGT CTC ACC ATC GAC TAT GC |  |
| Lrp6 reverse | AAG GAT GAG GCA AGT CAT CTG |  |
